# Production and Characterization of Lipases by Two New Isolates of *Aspergillus* through Solid-State and Submerged Fermentation

**DOI:** 10.1155/2015/725959

**Published:** 2015-06-09

**Authors:** Luciane Maria Colla, Aline M. M. Ficanha, Juliana Rizzardi, Telma Elita Bertolin, Christian Oliveira Reinehr, Jorge Alberto Vieira Costa

**Affiliations:** ^1^Laboratory of Fermentations, Food Engineering Course, University of Passo Fundo, Campus I, km 171, BR 285, P.O. Box 611, 99001-970 Passo Fundo, RS, Brazil; ^2^Laboratory of Biochemical Engineering, College of Chemistry and Food Engineering, Federal University of Rio Grande do Sul, P.O. Box 474, 96203-900 Rio Grande, RS, Brazil

## Abstract

Due to the numerous applications of lipases in industry, there is a need to study their characteristics, because lipases obtained from different sources may present different properties. The aim of this work was to accomplish the partial characterization of lipases obtained through submerged fermentation and solid-state fermentation by two species of *Aspergillus*. Fungal strains were isolated from a diesel-contaminated soil and selected as good lipases producers. Lipases obtained through submerged fermentation presented optimal activities at 37°C and pH 7.2 and those obtained through solid-state fermentation at 35°C and pH 6.0. The enzymes produced by submerged fermentation were more temperature-stable than those obtained by solid-state fermentation, presenting 72% of residual activity after one hour of exposition at 90°C. Lipases obtained through submerged fermentation had 80% of stability in acidic pH and those obtained through solid-state fermentation had stability greater than 60% in alkaline pH.

## 1. Introduction

Lipases (triacylglycerol acyl-hydrolases EC3.1.1.3) are enzymes capable of hydrolyzing the ester bonds of insoluble substrates in water at the substrate-water interface [1]. The main industrial applications of lipases are in detergents [2], medicines [3], and foods [1]. The maturation of cheeses [4], the synthesis of aromas [5], and the production of lipids with high levels of unsaturated fatty acids [6, 7] are examples of the application in food industry. The production of methyl-esters of fatty acids (biodiesel) [8] is the most recent and mentioned application nowadays.

As revised by Treichel et al. [9] many researchers worldwide direct their activities to the screening of new lipase-producing microorganisms and, subsequently, to the optimization of the medium composition and operational variables. All these efforts are justified by the great versatility of lipase applications. Due to the numerous applications of lipases in industry, there is a need to study their characteristics, because lipases obtained from different sources may have different properties [10].

The optimum activity of enzymes depends on the integrity of its structure; therefore factors that may affect these, such as pH, temperature, chemical agents, autolysis (proteases), and ionic strength, affect the enzyme's maximum activity [11]. Temperature has a significant effect on the kinetic energy of enzyme molecules and substrates and causes a greater number of productive collisions per unit of time. The inactivation of enzymatic activity can result from the absorption of excessive energy which causes the disruption or denaturation of the enzyme's tertiary structure due to changes in bonds, such as hydrogen bonds, disulfide bonds, and hydrophobic interactions [12]. The pH affects the stability of enzymes by changing the electrostatic interactions of their protein structure, causing changes in the amino acids' ionization status, which defines the secondary and tertiary structures of protein and therefore its activity and stability [13, 14].

According to Glogauer et al. [15], determination of the pH stability of enzymes is important for identifying nondenaturing pH values of buffers for purification, storage, and reaction steps. With regard to temperature, in an enzymatic process there is a critical play between thermostability and the effect of temperature on activity. It is necessary to identify a reaction temperature that at the same time allows a reasonably high rate of reaction and keeps the rate of denaturation at a reasonably low level.

Given the importance of characterizing lipases obtained from new sources in order to determine their application, the aim of this work was to characterize the lipases produced by* Aspergillus flavus* and* Aspergillus niger* through submerged and solid-state fermentation, respectively, according to the optimum temperature and pH, and to determine the stability of enzymes in relation to temperature and pH.

## 2. Material and Methods

### 2.1. Microorganisms: Isolation, Maintenance, and Inoculum Preparation

The filamentous fungi* Aspergillus* (strain O-8) and* Aspergillus* (strain O-4) were isolated from diesel-contaminated soil and previously selected as a good producer of lipase through submerged [16] and solid-state fermentation [17], respectively. The contaminated soil was collected after a case of leaking diesel from a storage tank to the fuel station, which occurred in the city of Passo Fundo, RS, Brazil.

The isolates were submitted to genetic identification through Phred/Phrap and Consed, using the methodology cited by Smaniotto et al. [18], at the Center of Nuclear Energy in Agriculture (Cena) from University of São Paulo (USP), Brazil. Sequences were compared to 18S rRNA data obtained from GenBank (http://www.ncbi.nlm.nih.gov/).

The isolate O-8 was identified as* Aspergillus flavus* strain DAOM (99% identity, GenBank accession number: JN938987.1) and the isolate O-4 was identified as* Aspergillus niger* DAOM (100% identity, GenBank accession number: KC545858.1).

After isolation, the microorganisms were kept in tubes with potato dextrose agar (PDA) inclined under 4°C refrigeration, with periodic replications every 3 months.

The inoculum preparation of* Aspergillus flavus* to submerged fermentation was carried out by inoculation of the fungi in Petri dishes with 30 mL of solidified PDA and incubation at 30°C for 5 days. The inoculation was accomplished using 10 mm of diameter circular areas containing spores growth prepared in Petri dishes [19].

The inoculum preparation of* Aspergillus niger* to solid-state fermentation was carried out by inoculating the fungus in 1 L Erlenmeyer's flasks containing 30 mL of solidified PDA medium and incubated at 30°C for 5 days. A spore suspension was obtained by adding 20 mL of a 0.1% Tween to the inoculum after incubation and by scraping the spores with a Drigalski loop. The fermentation media were inoculated with 2.10^6^ spores/g [19].

### 2.2. Culture Medium to Solid-State and Submerged Fermentation

The culture conditions of lipase production in submerged and solid-state fermentation had been previously optimized [19].

The medium to submerged fermentation was prepared with 10% (w/v) of wheat bran, which was boiled at 100°C for 30 min. Afterwards, the medium was filtered and the soluble extract added to 10% (v/v) of saline solution, 45 g/L of yeast extract as nitrogen source and 20 g/L of soybean oil as inducer. The composition of saline solution [20] was 2 g/L KH_2_PO_4_, 1 g/L MgSO_4_, and 10 mL/L of trace solution containing (mg/L) FeSO_4_·7H_2_O (0.63), MnSO_4_ (0.01), ZnSO_4_  (0.62). The medium was autoclaved at 103 kPa for 20 min and the pH adjusted to 7.0 using HCl 1.5 mol/L or NaOH 1 mol/L. After inoculation, the cultures were incubated for 4 days at 30°C with agitation of 160 min^−1^.

The medium for solid-state fermentation was prepared under previously optimized conditions [20] with 85% of soybean or wheat bran and 15% of rice husk. The medium was added to 71% (v/w) of saline solution [20] and 2% of sodium nitrate as nitrogen source. The medium was autoclaved at 103 kPa for 20 min and subsequently added to 2% olive oil as an inducer of lipase production. The pH was adjusted to 4.5 by the addition of a 1.5 mol/L solution of H_2_SO_4_ and moisture was adjusted to 60% by adding sterile distilled water. Fermentations were carried out in 300 mL Erlenmeyer's flasks containing 50 g of the medium, which were incubated at 30°C for 96 h after inoculation. The fermented brans were kept at −20°C until use.

### 2.3. Achievement of Enzymatic Extracts

After the production of lipase by submerged and solid-state fermentation, procedures for obtaining the enzymatic extracts were conducted, which are described below.

The fermented medium obtained under submerged fermentation by the fungi* Aspergillus flavus* was filtered in cotton for the retention of hyphae and frozen at −20°C, being after used in the determinations of enzymatic activities [19].

The extraction of lipase from the fermented bran obtained in solid-state fermentation by the fungi* Aspergillus niger* was carried out by adding 10 mL buffer with pH established in each methodology at 1 g of fermented medium, followed by agitation of 160 min^−1^ for 30 min at 37°C. The extract was cotton-filtered and used as enzyme extract in subsequent reactions [19].

### 2.4. Effect of pH and Temperature on the Optimal Activity of Enzymatic Extracts

The enzymatic extracts without the cells of microorganisms were submitted to tests to determine the influence of pH and temperature on the enzymatic activities.

The optimum activity of enzymatic extracts produced through submerged fermentation was determined by a Central Composite Design (CCD) composed of 4 factorial points, 4 axial points, and 3 central points (Table 1). The levels of variables ranged from 28°C to 42°C for temperature and 6.3 to 7.7 for pH. Optimum pH and temperature for activity of the enzymes produced through solid-state fermentation were determined using a 3^2^ Full Factorial Design (FFD) (Table 2). The variables levels were 5 to 7 for the pH and 30°C to 40°C for the temperature.

The enzyme activity was determined using the method standardized by Burkert et al. [21] which is based on titration with NaOH of fatty acids released by the action of lipase in the extract on the triacylglycerols of olive oil emulsified in arabic gum. The following were added to 250 mL flasks: 2 mL buffer prepared according to the objective of the test, 5 mL of emulsion prepared with 75 mL of 7% arabic gum, and 25 mL of olive oil. Next, 1 mL of enzyme extract was added to this system and it was incubated at temperatures described in the experimental design for 30 min. After incubation, the reaction was stopped by adding 15 mL of acetone : ethanol : water (1 : 1 : 1) and the released fatty acids were titrated with a solution of 0.01 mol/L NaOH using phenolphthalein as indicator. One unit of activity was defined as the amount of enzyme that releases 1 *μ*mol of fatty acid per minute per mL of enzyme extract of submerged fermentation (1 U = 1 *μ*mol/min·mL) or per g of fermented brand (1 U = 1 *μ*mol/min·g) of solid-state fermentation, under the test conditions.

### 2.5. Temperature Stability of Enzymatic Extracts

Thermostability of lipases obtained through submerged and solid-state fermentation was measured by incubating the enzyme extract at 35°C to 90°C. Aliquots were periodically taken to measure lipolytic activity, using the optimum temperature and pH for enzyme activity, obtained as mentioned in Section 2.4, to each enzymatic extract. The experiments were duplicated.

For the enzymes obtained through solid-state fermentation it was possible to calculate the Arrhenius thermal deactivation and activation energy for thermal destruction constants (*E*_*a*_). Therefore, the data of enzymatic activity at each temperature tested were used to calculate the residual lipase activity (*RA*) over time. The constant of thermal deactivation (*k*_*d*_) at each temperature was calculated by linear regression of the data of Ln (*RA*) versus time, according to the Arrhenius kinetic model, considering that inactivation of the enzyme obeys first-order kinetics, as in the following. Consider

(1) $$\begin{array}{c} \frac{d\left[E\right]}{\left[E\right]}=-k_{d}\cdot dt. \end{array}$$

After integration,

(2) $$\begin{array}{c} Ln\frac{{\left[E\right]}_{2}}{{\left[E\right]}_{1}}=-k_{d}\cdot \Delta t. \end{array}$$

Considering that the enzyme concentration ([*E*]) is directly proportional to the enzymatic reaction speed,

(3) $$\begin{array}{c} \frac{{\left[E\right]}_{2}}{{\left[E\right]}_{1}}=AR. \end{array}$$

We get the following:

(4) $$\begin{array}{c} Ln\left(AR\right)=-k_{d}\cdot \Delta t. \end{array}$$

From the thermal deactivation constants at each temperature, the half-lives (*t*_1/2_) were obtained (5) which corresponds to the time required, at the temperature tested, so that 50% of the initial enzyme concentration is inactivated:

(5) $$\begin{array}{c} t_{1/2}=\frac{0.693}{k_{d}}. \end{array}$$

The activation energy (*E*_*a*_) for thermal destruction of the enzyme was calculated from (6). The value of *E*_*a*_ was obtained from the inclination of the regression line of ln *K* versus 1/*T*:

(6) $$\begin{array}{c} lnK=lnA-\frac{E_{a}}{RT}, \end{array}$$

where [*E*] = enzyme concentration, *AR* = residual activity of the enzyme, *t* = time (min), *k*_*d*_ = thermal deactivation constant,* A* = Arrhenius factor (depending, among other things, on the contact area), *E*_*a*_ = activation energy,* R* = ideal gases constant (8.314 J mol^−1^K^−1^), and* T* = absolute temperature (K).

### 2.6. pH Stability of Enzymatic Extracts

The effect of pH on the stability of enzymes obtained through submerged fermentation was determined by treating 1 mL of enzyme extract with 2 mL of buffer solutions at pH 3.5, 4.0, 4.5, 5.0, 5.5, 6.0, 6.5, 7.0, 8.0, and 9.0 for 24 hours at 25°C. The buffers used were 0.1 mol/L citrate (pH 3.5), 0.2 mol/L acetate (pH 4.0 to 5.5), 0.2 mol/L phosphate (pH 6.0 to 8.0), and 0.2 mol/L glycine (pH 9 and 10). Enzyme activity in initial and final times was carried out at optimum temperature and pH for enzyme activity, obtained from the results of the assays of Section 2.4. The experiments were duplicated.

The stability of enzymes obtained through solid-state fermentation was assessed through their extraction from the fermented medium using the following buffer solutions: 0.1 mol/L citrate (pH 3.5), 0.2 mol/L acetate (pH 4.0, 4.5, 5.0, and 5.5), 0.2 mol/L phosphate (pH 6.0, 6.5, 7.0, 7.5, and 8.0), and 0.2 mol/L glycine (pH 9 and 10). The extracts were kept at 25°C for 24 h and the residual lipolytic activity was determined at optimum temperature and pH for enzyme activity, according to the results obtained from the assays of Section 2.4.

## 3. Results and Discussion

In the submerged culture fermentation the microorganisms grow in a liquid medium in which the nutrients are dissolved. In solid-state fermentation the microorganisms grow on the surface of a solid matrix in which the nutrients are adsorbed, and the moisture does not exceed the water retention capacity of this matrix [22, 23]. These differences between production methods as well as the differences between the microorganisms used in fermentation processes can lead to obtaining lipases with different characteristics.

### 3.1. Effect of pH and Temperature on the Optimal Activity of Enzymatic Extracts

The pH and temperature have great influence on the enzyme activity, being important to define these parameters for the characterization of the enzymes obtained. After fungal growth in culture media of submerged and solid-state fermentations, enzymatic extracts were obtained as described in Section 2.3 and used in the assays mentioned in Section 2.4. The results of enzymatic activities were presented in Tables 1 and 2, which also shows the experimental conditions of the experimental designs used to determine optimum temperatures and pH of the enzymes produced through submerged and solid-state fermentations, respectively.

The highest lipolytic activities in the solid-state fermentation (Table 2) may be due to the characteristics of this type of cultivation when compared with submerged cultivation. In the solid state fermentation, the concentration of the final product is higher and the fungus has the appropriate characteristics, as tolerance to low water activity and production of enzymes through hyphae [23]. Furthermore, lipase production was performed by different microorganisms, although both are of the genus* Aspergillus*.

The analysis of variance of lipolytic activity obtained in each experimental design demonstrated, that in both cases, the *F*_calculated_ obtained in the analysis of regression models were higher than the *F*_tabulated_ value (*F*_calculated_ of 6.43 and *F*_tabulated_ of 2.85 for Composite Central Design of submerged fermentation; *F*_calculated_ of 32.2 and *F*_tabulated_ of 2.80 for Full Factorial Design of solid-state fermentation), which means that the variation caused by the models is significantly greater than the unexplained variation [24].

Equations (7) and (8) show the regression models for the enzymes obtained through submerged fermentation (SmF) and solid-state fermentation (SSF), respectively. The correlation coefficients between experimental data and models were of 81.7% and 94.02%, which validates the mathematical models obtained [25, 26]:

(7) ALSmF=4.19+0.28·X1−0.254·X12+0.142·X2 −0.17·X22,

(8) $$\begin{array}{c} {AL}_{SSF}=49.2+2.7\cdot X_{1}-17.7\cdot {X_{1}}^{2}-14.9\cdot {X_{2}}^{2}. \end{array}$$

The estimated effects of variables of CCD on lipolytic activity showed that linear and quadratic effects of pH were significant (*P* < 0.05). The temperature had significance levels very close to 0.05, of *P* = 0.057 and *P* = 0.054, for the linear and quadratic effects, respectively. Thus, the effect of temperature was considered for the expression of the model. Both variables showed positive linear effects, and the effect of pH was greater than the effect of temperature (0.561 and 0.284, resp.).

Linear and quadratic effects of pH were significant (*P* = 0.02 and *P* < 0.01, resp.) and positive on the activity of lipases obtained through solid-state fermentation (5.4 and −35.4, resp.). On the other hand, only the quadratic effect of temperature (−29.7) had significant influence (<0.01) on the lipolytic activity.

The use of response surface methodology has the advantage of allowing the estimation of the effects of experimental variables on the response variable within the limits stipulated by the experimental design. Thus, the optimal values of the experimental variables can be calculated from the mathematical modeling of the data, when the response surface is validated by analysis of variance. The optimal pH and temperature were calculated by equating the first derivative of the lipolytic activity as a function of the pH and temperature to zero in both mathematical models. The maximum lipolytic activities of lipases obtained through submerged fermentation were obtained at the levels +0.55 and +0.417 from pH and temperature, respectively, corresponding to pH 7.2 and temperature of 37°C. The maximum lipolytic activities of lipases obtained through solid-state fermentation were obtained at the levels +0.076 and zero (0) to pH and temperature, respectively, corresponding to pH 6.0 and temperature of 35°C. These results could differ a little from the maximum results obtained in Tables 1 and 2, because they were obtained from the mathematical models generated from the experimental data.

**Figure 1:**
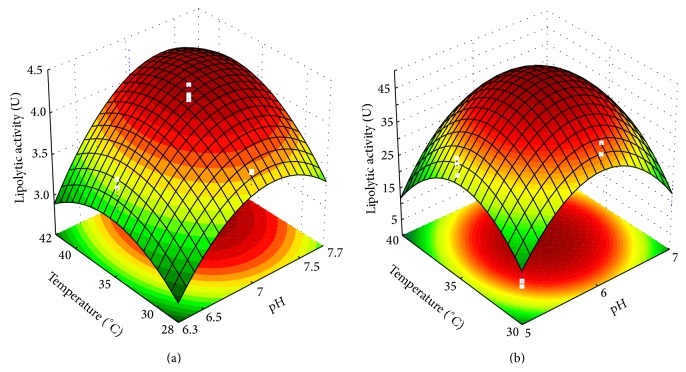
Response surface of the influence of pH and temperature on the lipolytic activity of enzymes produced (a) through submerged fermentation by* Aspergillus flavus* (strain O-8) from CCD and (b) through solid-state fermentation by* Aspergillus niger* (strain O-4) from the 3^2^ Full Factorial Design.

Figures 1(a) and 1(b) show the response surface representing the mathematical models of (7) and (8) of the enzymes obtained through submerged and solid-state fermentation, respectively.

The optimum pH and temperature found in this study for the activity of fungal lipases are similar to results reported in academic literature, in which maximum lipase activities were obtained at around 30°C and 40°C and pH 6.0 to 8.0 [27].

Maldonado [10] obtained maximum lipolytic activities at pH 7.0 and 37°C for crude and purified lipase of* Geotrichum candidum*. Baron et al. [28] found that there is a pH range (6.0 and 8.0) where enzyme activity is maximal and that the activity depends not only on the pH of the medium but also on the type of buffer used. Freire et al. [29] reported that the optimum pH and temperature for lipases produced by* Penicillium* sp. were 7.0 and 37°C. Benjamin and Pandey [30] reported that the lipases produced by* Candida rugosa* showed optimum activity at pH 7.0 and temperature 40°C. Pastore et al. [31] characterized the lipases produced by* Rhizopus* sp. and found maximum activity at pH 6.0 and 6.5 and 40°C. However, Diaz et al. [32] characterized the lipases of the fungus* Rhizopus homothallicus* obtained by solid-state and submerged bioprocesses, obtaining maximum activity at pH 7.5 and 30 to 40°C, respectively.

Santos et al. [33] and Lotrakul and Dharmsthiti [34] characterized lipases that obtained maximum activities at temperatures higher than those obtained in this study: 45°C to 50°C for the lipase produced in solid-state fermentation by* Trichosporon* spp., and 45°C for the lipase from* Aeromonas sobria* isolated from raw milk, respectively. Shangguan et al. [35] studied a lipase obtained from* A. fumigatus* that presented optimum pH and temperature of 8.5 and 65°C, respectively.

### 3.2. Temperature Stability

These experiments were conducted to evaluate the stability of enzymes obtained through two fermentation processes. After maintenance of enzymatic extracts at temperatures ranging from 35°C to 90°C for different periods of time (Section 2.5), the enzymatic activity was evaluated at pH and temperatures optimized in the previous step (Section 2.4) to each fermentation process.

**Figure 2:**
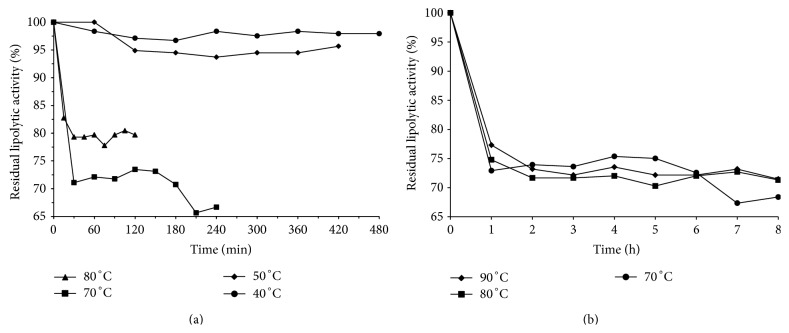
Thermal stability of lipases produced in submerged fermentation using* Aspergillus flavus* (strain O-8): (a) 40°C, 50°C, 70°C, and 80°C; (b) 70°C, 80°C, and 90°C.

The temperature stability of lipases produced through submerged fermentation by* Aspergillus flavus* was initially assessed at 40 to 80°C, as shown in Figure 2(a). The enzymes were stable between 40°C and 50°C, with residual activity greater than 90% for 7 hours. An initial inactivation of the enzymes between 70°C and 80°C was observed and subsequently they became stable, with residual activity of 80% and 65% at 80°C and 70°C, respectively. The enzymes did not exhibit first-order thermal destruction kinetic behavior (Arrhenius), despite being more stable at 80°C than 70°C.

In order to confirm the data obtained, tests were carried out at 70°C, 80°C, and 90°C for 8 h, and the results (shown in Figure 2(b)) confirm the aforementioned behavior, with an initial enzyme inactivation, caused by thermal shock, and later stability. Furthermore, the enzymes exhibited greater stability at higher temperatures in the first hour of testing. The lipases were stable at 70°C to 90°C, with mean residual activities of around 72% after the first hour of incubation.

**Figure 3:**
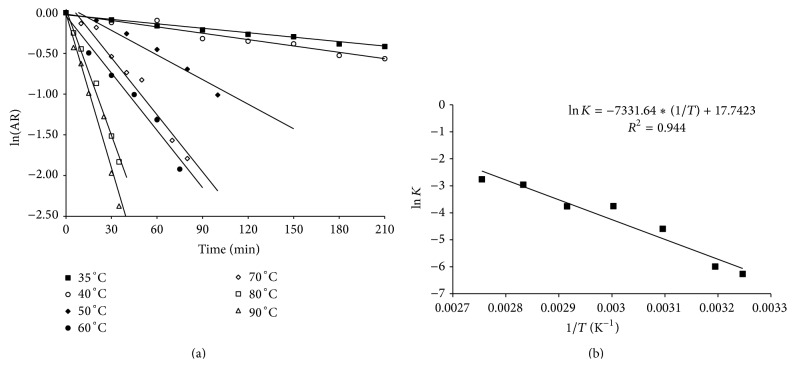
Kinetics of thermal destruction of the enzymatic extracts produced by* Aspergillus niger* in solid-state fermentation: (a) at temperatures of 35°C to 90°C. *AR*: enzyme residual activity, (b) linear regression of the thermal deactivation constants obtained at 35°C to 90°C (ln of data) as function of inverse of absolute temperature for calculating the energy of thermal deactivation of the enzyme.

Figure 3(a) shows the thermal destruction kinetics of the enzyme produced through solid-state fermentation between 35°C and 90°C, which follows the pattern of first-order thermal destruction predicted by the Arrhenius model.  Table 3 shows the thermal deactivation constants between 35°C and 90°C, obtained from angular coefficients of the curves shown in Figure 3, as well as the determination coefficients of regression and the half-life of enzymes at each temperature. The enzyme had higher thermal stability at 35°C and 40°C, which can be observed from the high half-lives (*t*_1/2_), around 6 and 4.3 h, respectively. Above 50°C, the half-life considerably decreased to 29 min between 60°C and 70°C.  Figure 3(b) shows the graph of ln (*k*_*d*_) as a function of absolute temperature (K), used to calculate the energy of thermal deactivation, which was 60.33 kJ/mol for the studied enzyme.

Lipase obtained with solid-state fermentation showed higher energy of deactivation than those obtained by Diaz et al. [32] and Lima et al. [36], which were 30 and 34.2 kJ/mol for the lipases produced by* Rhizopus homothallicus* and* Bacillus megaterium*, respectively. However, they had lower deactivation energy than the ones obtained by Maldonado [10], which were 330, 140, and 182 kJ/mol for the lipases produced by* Geotrichum candidum* in media containing peptone, hydrolyzed yeast, and macerated clarified corn water, respectively. The activation energy reflects the dependence of the thermal deactivation constant with respect to temperature [37, 38], and the higher the constant, the greater the variation of the thermal deactivation constant with the temperature variation.

Razak et al. [39] reported that fungal lipases in general are unstable above 40°C, with moderate stability, contrary to what has been observed in lipases produced by bacteria such as* Bacillus* [40, 41] and* Pseudomonas* [42], which are thermostable above 60 C. However, the lipases produced by* Rhizopus* sp. maintained 50% or more of their activity when heated for 60 min between 40 and 55°C [31] and lipases produced by Geotrichum-like R59 showed thermostability, with maximum residual activity after incubation at 60°C for 1 h [43].

Shu et al. [44] reported that the lipases produced by* Antrodia cinnamomea* had 50% residual activity between 25°C and 40°C. Lipase from* Aspergillus niger* NCIM 1207 was stable at 40°C for 3 h; however the treatment at 50°C for 1 h caused 52% loss of activity [45]. Baron et al. [28] showed that the lipases produced by* Penicillium corylophilum* were completely inactivated after 30 min at 60°C. However, the lipases produced by* Rhizopus* sp. had moderate thermostability, with 70% residual activity at temperatures of 40 to 55°C [31]. Ginalska et al. [43] showed that the lipases produced by* Geotrichum* sp. had 100% residual activity after 1 hour of incubation at 60°C, and 50% residual activity at 70°C for 45 min. Furthermore, Sharma et al. [14] reported that the lipase produced by* Bacillus* sp. RSJ-1 presented 90 and 70% residual activity after treatment at 50°C for 120 and 240 min, respectively. Lipase obtained from Iftikhar et al. [46] showed that lipases retained 80% of its activity at 25–30°C by wild and 100% of its activity at 20–50°C by mutant strain of* R. oligosporus*. By further increase in the incubation temperature, the activity of the enzyme was greatly inhibited.

Compared with the aforementioned enzymes, lipases obtained in this study through submerged fermentation had higher thermostability and may have applications in industrial processes that require high temperatures. Enzymatic processes that occur at higher temperatures have higher reaction rates [11]. It may be possible to use thermostable lipases in the synthesis of biopolymers, pharmaceuticals, agrochemicals, cosmetics, biodiesel, and aromas [47]. According to Diaz et al. [32] even for identical lipases produced by different methods of cultivation (submerged and solid-state), there may be thermostability differences caused by the binding of nonprotein compounds derived from the culture medium through noncovalent bonds to the lipases, changing their physical and chemical properties.

Enzyme thermostability may be affected by production conditions, such as the producer microorganism, the method of cultivation, and the medium used [10]. Thermostability is the result of the protein's amino acid sequence, which provides a more rigid conformation to the enzyme [11] through intramolecular interactions, with the internalization of hydrophobic residues and superficial exposure of hydrophilic residues [45]. Lipase thermostability may also be affected by the presence of compounds such as short-chain alcohols, metals, and ions as Ca^+2^ and Mg^+2^ which bind to the surface of enzymes whose binding sites are generally formed by negatively charged groups [48]. According to Iyer and Ananthanarayan [11] thermal stabilization of lipases may be caused by the presence of divalent ions, anions (SO_4_^−2^ > Cl^−^ > Br^−^ > NO_3_^−^ > ClO_4_^−^), or cations (NH_4_^+^ > K^+^ > Na^+^ > Mg^+2^ > Ca^+2^ > Ba^+2^). Mg^+2^ and SO_4_^−2^ were present in the lipase production culture medium, which, if not consumed by the fungus for growth and synthesis, remain soluble after the separation of cells, and become part of the lipolytic extract. That may explain the thermostability of the produced enzymes. However, if this enzyme extract containing lipases were purified for further use, causing the removal of these ions of the culture medium, the study of the stability of the purified protein would be needed.

### 3.3. pH Stability

The pH stability of enzymatic extracts obtained through submerged and solid-state fermentation was determined according to Section 2.6 treating these extracts with different buffers for 24 h and after the enzymatic activity was determined using the optimized pH and temperature for the enzymes of each fermentation process (Section 2.4).

**Figure 4:**
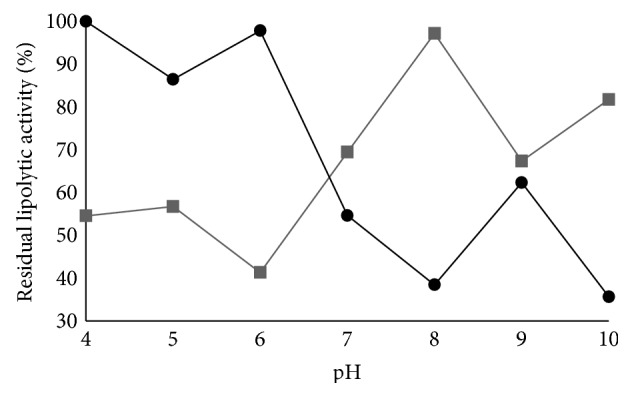
pH stability of lipases produced (●) by* Aspergillus flavus* (strain O-8) through submerged fermentation and (■) by* Aspergillus niger* (strain O-4) through solid-state fermentation.

Figure 4 shows the residual lipolytic activity as a function of pH for the enzymes produced through solid-state and submerged fermentation. Lipases produced through submerged fermentation by* Aspergillus flavus* were stable at pH ranging from 3.5 to 6.5 for 24 h, with residual activities greater than 80%. At pH 7 to 10 there was a reduction in the stability of enzymes with residual activity of around 50%.

Lipase produced through solid-state fermentation by* Aspergillus niger* had greater stability at pH greater than 7.0, with residual activity greater than 60%. In acidic pH (4 to 6), the stability of the enzyme after 24 h was around 50%. It was found that the enzyme showed optimal activity at acidic pH (6.0), while the highest stability was observed with alkaline pH.

This behavior is similar to that reported by Mhetras et al. [45], who reported that lipases produced by* Aspergillus niger* NCIM 1207 were stable when pH was alkaline (pH 8 to 11) despite having had optimum activity at an acidic pH. Sharma et al. [14] reported that the lipases produced by* Bacillus* sp. RSJ-1 had 84 and 82% residual activity, respectively, after 2 h at pH 8 and 9. The lipases produced by* Candida* sp. were stable at pH ranging from 7.5 to 8.5 for 15 min [27].

## 4. Conclusion

Lipase produced by the* Aspergillus flavus* (strain O-8) through submerged fermentation had maximum activities at 37°C and pH 7.2. The thermal stability was 72% after 1 h of exposure to temperatures of 70 to 90°C and pH stability greater than 80% in acidic pH, which are desirable traits for industrial application. On the other hand, lipases produced through solid-state fermentation with* Aspergillus niger* (O-4) had optimum temperature and pH around 35°C and pH 6.0 and stability at room temperature (63.6% and 26.8% of residual activity after 1 h of exposure to 50 and 60°C, resp.), lower than that observed with enzymes obtained through submerged fermentation. The pH stability was higher in alkaline pH, with residual activity greater than 60% after 24 h of exposure.

## Figures and Tables

**Table 1:** Central composite design (CCD) used to determine the influence of pH and temperature on the optimal activity of lipase obtained through submerged fermentation by *Aspergillus flavus* (strain O-8) and results of lipolytic activity (U/mL). Results of mean and standard deviation.

Experiment	pH (*X*_1_)^*^	Temperature (*X*_2_)	Lipolytic activity (U/mL)
1	6.5 (−1)	30°C (−1)	3.15 ± 0.13
2	7.5 (+1)	30°C (−1)	3.72 ± 0.01
3	6.5 (−1)	40°C (+1)	3.21 ± 0.07
4	7.5 (+1)	40°C (+1)	4.02 ± 0.08
5	6.3 (−1.414)	35°C (0)	3.54 ± 0.05
6	7.7 (+1.414)	35°C (0)	4.23 ± 0.02
7	7.0 (0)	28°C (−1.414)	3.82 ± 0.01
8	7.0 (0)	42°C (+1.414)	4.37 ± 0.03
9	7.0 (0)	35°C (0)	4.30 ± 0.12
10	7.0 (0)	35°C (0)	4.26 ± 0.01
11	7.0 (0)	35°C (0)	4.03 ± 0.03

^*∗*^Conditions of pH obtained using 0.2 M phosphate buffer in the enzymatic activity determination.

**Table 2:** Full Factorial Design (3^2^) used to determine the influence of pH and temperature on optimum activity of lipase obtained through solid-state fermentation by *Aspergillus niger* (strain O-4) and results of lipolytic activity (U/g). Results of mean and standard deviation.

Experiment	pH (*X*_1_)^*^	Temperature (°C) (*X*_2_)	Lipolytic activity (U/g)
1	5 (−1)	30 (−1)	12.20 ± 0.96
2	6 (0)	30 (−1)	40.62 ± 1.90
3	7 (+1)	30 (−1)	15.49 ± 1.92
4	5 (−1)	35 (0)	34.14 ± 2.52
5	6 (0)	35 (0)	42.82 ± 1.65
6	7 (+1)	35 (0)	35.41 ± 1.92
7	5 (−1)	40 (+1)	10.53 ± 0.96
8	6 (0)	40 (+1)	34.58 ± 1.65
9	7 (+1)	40 (+1)	22.13 ± 0.96

^*∗*^Conditions of pH obtained using 0.2 M phosphate buffer in the steps of enzyme extraction of the fermented bran and in the enzymatic activity determination.

**Table 3:** Thermal deactivation constant (*k*_*d*_), correlation coefficients of regressions (*R* ^2^), and times of half-lives (*t*_1/2_) of enzymatic extracts of solid-state fermentation at temperatures of 35°C to 90°C.

Temperature (°C)	*k*_*d*_ (min^−1^)	*R* ^2^	*t*_1/2_ (min)
35	0.0019	0.985	364.74
40	0.0027	0.954	256.67
50	0.0100	0.966	69.30
60	0.0234	0.976	29.62
70	0.0233	0.971	29.74
80	0.0516	0.991	13.43
90	0.0630	0.968	11.00
